# Patients Who Die by Suicide: A Study of Treatment Patterns and Patient Safety Incidents in Norway

**DOI:** 10.3390/ijerph191710686

**Published:** 2022-08-27

**Authors:** Sanja Krvavac, Billy Jansson, Ida Rashida Khan Bukholm, Rolf Wynn, Martin Bystad

**Affiliations:** 1 Department of Psychiatry, Helgeland Hospital Trust, 8802 Sandnessjøen, Norway; 2 Department of Health and Care Sciences, UiT The Arctic University of Tromsø, 9037 Tromsø, Norway; 3 Department of Psychology and Social Work, Mid Sweden University, 831 25 Ostersund, Sweden; 4 The Norwegian System of Patient Injury Compensation, 0130 Oslo, Norway; 5 Faculty of Landscape and Society, The Norwegian University of Life Sciences, 1430 Ås, Norway; 6 Department of Clinical Medicine, UiT The Arctic University of Tromsø, 9038 Tromsø, Norway; 7 Division of Substance Use and Mental Health, University Hospital of North Norway, 9019 Tromsø, Norway

**Keywords:** suicide, patients, treatment, patient safety, medical errors

## Abstract

Underlying patterns and factors behind suicides of patients in treatment are still unclear and there is a pressing need for more studies to address this knowledge gap. We analysed 278 cases of suicide reported to The Norwegian System of Patient Injury Compensation, drawing on anonymised data, i.e., age group, gender, diagnostic category, type of treatment provided, inpatient vs. outpatient status, type of treatment facility, and expert assessments of medical errors. The data originated from compensation claim forms, expert assessments, and medical records. Chi-square tests for independence, multinominal logistic regression, and Bayes factors for independence were used to analyse whether the age group, gender, diagnostic category, inpatient/outpatient status, type of institution, and type of treatment received by patients that had died by suicide were associated with different types of medical errors. Patients who received medication tended to be proportionally more exposed to an insufficient level of observation. Those who received medication and psychotherapy tended to be proportionally more exposed to inadequate treatment, including inadequate medication. Inpatients were more likely to be exposed to inappropriate diagnostics and inadequate treatment and follow up while outpatients to insufficient level of observation and inadequate suicide risk assessment. We conclude that the patients who had received medication as their main treatment tended to have been insufficiently observed, while patients who had received psychotherapy and medication tended to have been provided insufficient treatment, including inadequate medication. These observations may be used as learning points for the suicide prevention of patients in treatment in Norwegian psychiatric services.

## 1. Introduction

Approximately 800,000 people die each year by suicide. This is the second leading cause of death among those aged 15 to 29 globally [1]. The complex dynamics of suicide risk factors involve suicidal behaviour, the history of suicide attempts, diagnosis, psychosocial factors, biological factors, and physical illness [2,3]. Between 2008–2015, 199 cases of suicide during treatment at Norwegian psychiatric institutions were reported to the Norwegian Patient Registry, i.e., approximately 25 patients per year [3].

There is a higher risk of suicide for inpatients in psychiatric institutions compared to a primary healthcare population [2,4,5]. Predictors of suicide among inpatients in psychiatric institutions include personality disorders and particularly borderline personality disorder, substance abuse, previous suicide attempt, and insufficient treatment or follow-up [4]. Furthermore, as increased suicidality is one important reason for involuntary hospitalisation, being involuntarily admitted is associated with an increased risk for suicide [6,7,8], especially during the first week of hospitalisation [9,10].

Psychiatric units should ideally be able to guarantee a high degree of patient safety. However, safety must be balanced against other factors that are of importance to patients’ treatment and integrity, such as patients’ freedom [11]. In addition, medical errors may diminish the institutions’ ability to protect patients [12]. Marcus and colleagues [13] found that one in five patients in psychiatric settings experienced a medical error or adverse event, and 56.6% of all adverse events were conceptualised as preventable. Medical errors, including assessment shortage (e.g., the undocumented/incomplete assessment of suicide risk or lack of follow up of corresponding assessments), safety measures failures (e.g., that the patient had access to dangerous objects/drugs), along with resource problems (e.g., the lack of a doctor/psychologist and/or insufficiently trained healthcare professionals) may increase the risk of suicide [14]. Other risk factors are long delays in diagnostics and treatment [15,16,17], diagnostic and medication errors, seclusion, harm from the use of restraints, and errors in the treatment of self-harm behaviours [18]—suggesting that patients’ need for adequate clinical suicide risk assessment and medicine are not met [19,20]. Importantly, the contribution of medical errors to suicidal behaviour has not been sufficiently explored in previous research.

The current study involves suicides reported to The Norwegian System of Patient Injury Compensation (Norsk Pasientskade Erstatning—NPE [21]). The suicides took place while the study participants were either inpatients or accessing outpatient treatment. A retrospective chart review was conducted. We examined whether the age group, gender, diagnostic category, inpatient/outpatient status, type of institution, and type of treatment received by patients that had died by suicide were associated with different types of medical errors.

## 2. Materials and Methods

### 2.1. Participants and Procedures

We examined data from The Norwegian System of Patient Injury Compensation [21] drawing on compensation claim forms, psychiatric case records, and expert assessments in the 10-year period (2009–2019) for 278 individuals who died by suicide.

NPE is a government agency that focuses on patient safety and processes claims for compensation from patients or their family members (i.e., in those cases where the patient is deceased or otherwise unable to report a claim) who believe that the patients sustained injuries as a result of treatment at a public or private somatic or mental health institution. The role of NPE is to inspect whether patient safety requirements have been fulfilled by investigating the reported adverse events and the actions taken by the health care providers. When receiving a complaint, NPE obtains all relevant information about the case. A lawyer and a medical expert working with NPE will then investigate the medical and legal aspects and approve or deny compensation. If approved, compensation is determined on an individual basis, with respect to the applicant’s disability and economic loss. The same type of injury may consequently result in different amounts of compensation.

To qualify for compensation after a patient injury, three conditions must be met: the injury must be caused by treatment failure, the patient must have sustained a financial loss and/or a permanent medical impairment (or death), and, finally, the claim must be made within three years after the claimant (i.e., the patient or next-of-kin) ought to have understood the connection between the treatment and the injury. Two exceptions from these rules are when the outcome is significantly worse than one could expect, and after hospital-acquired infections. In these cases, compensation can be granted even if no health-service-related failure has taken place.

A selection of variables, including the patients’ age, gender, diagnosis (ICD-10), institution responsible for treatment, medical field, type of treatment/procedure, and type of injury caused by medical treatment failure, are registered by NPE staff in a structured manner in a data base, following a coding system. Data for the present study were extracted from this data base by NPE and subsequently anonymised and aggregated in an Excel file that was provided to the researchers for further analyses.

The inclusion criteria were: (1) that the patient had died by suicide, (2) that the patient had received inpatient or outpatient treatment in the period when the suicide occurred, (3) that a potential medical error had taken place, and (4) that a compensation claim had been made by next-of-kin on the NPE website [21]. We analysed all the cases reported by NPE that fulfilled the criteria. Data were analysed with non-parametric statistical methods in SPSS 27 [22] and Jeffreys’s Amazing Statistics Program—JASP [23].

The study was performed in accordance with the Declaration of Helsinki. All relevant guidelines and regulations were followed. The study was approved by the head of NPE and the Research Assessment Committee of NPE (Personvernombudet).

### 2.2. Measures

**Diagnosis.** Psychiatric ICD-10 [24] diagnoses (see Table 1) were extracted from the case records. These were grouped into three categories: (1) F20 Schizophrenia and psychotic disorders, (2) F30 Mood disorders, and (3) All other diagnoses, including substance use, anxiety disorders, eating disorders, personality disorders, etc.

**Patient status.** Patient status was extracted from the case records and coded as a dichotomous variable (inpatient/outpatient) indicating that the patient was either hospitalised at a psychiatric unit/on leave at the time of the suicide or received outpatient treatment.

**Type of health care.** The type of health care institution data was extracted from patients’ clinical case records and coded as a dichotomous variable: (1) psychiatric institutions in Norway where participants were treated and/or hospitalised and (2) other institutions (public general hospitals, community health care services, private practice health care specialists, general practitioners’ clinics, addiction treatment services, and private hospitals working for the public health services; this category also includes somatic units).

**Type and complexity of treatment.** Data on the type of treatment given were extracted from NPE expert evaluations and the patients’ electronic health records and were coded according to the complexity of treatment given, as follows: (1) medication, (2) medication and psychotherapy, and (3) Electroconvulsive therapy (ECT), medication, and psychotherapy.

**Types of medical errors.** This measure was extracted from NPE expert evaluations and coded as: (1) Insufficient level of observation of symptoms and lack of safety measures, which indicated that the unit’s personnel most of the time failed to observe all the patient’s symptoms; (2) Inadequate suicide risk assessment, was mostly based on staff being unqualified and/or inexperienced; (3) Insufficient/delayed diagnostic assessment implied that assessment was erroneous or significantly delayed; (4) Inadequate treatment, including inadequate medication, and insufficient follow up.

### 2.3. Interrater-Reliability Analysis

Interrater reliability analyses (IRR) of type of treatment, inpatient vs. outpatient, and types of medical errors were performed by a clinician (H.B.) who extracted and generated 30 random cases for the inpatient vs. outpatient variable with an overall agreement of 86% (Cohen’s Kappa = 0.86, *p* ˂ 0.001), along with 60 cases for the variables type of treatment with an overall agreement of 70% (Cohen’s Kappa = 0.72, *p* ˂ 0.001), and medical errors 90% (Cohen’s Kappa = 0.99, *p* ˂ 0.001).

### 2.4. Data Analytic Procedures

To examine the interrelation between the risk factor variables (sociodemographic, clinical, and institutional) and the medical errors variable (all being categorical variables), multinominal logistic regression was used in order to obtain Chi-square values, *p*-values, and effect sizes (odds ratios). In case of significant effects, post-hoc tests were conducted to explore the relative contribution of the cells (using contingency tables) by calculating the adjusted residuals (see Results for more details).

Bayes factors for Independence [25,26] were calculated (joint multinomial sampling with columns fixed) to determine the presence or absence of effects. Bayesian hypothesis testing determines the relative degree of evidence for or against the alternative hypothesis [27].

A Bayes factor (BF) is a numerical value quantifying how well a hypothesis predicts the empirical data relative to a competing hypothesis. BF_10_ expresses the likelihood of the data given the alternative hypothesis relative to the likelihood of the data given the null hypothesis. In this study, the BF was used to index the presence (evidence for H_1_ compared to H_0_) or absence of a difference (evidence for H_0_ compared to H_1_) between conditions. According to H_1_, clinical risk factors contributed to suicide risk, whereas H_0_ did not find any such relationship. For institutional factors, the H_1_ is that institutional factors affect suicide risk, while H_0_ states that these factors do not have an impact on suicide risk. For patient status, the H_1_ states that patient status affects suicide risk and H_0_ states that this is not the case. For type of health care, the H_1_ states that type of health care does have an impact on suicide risk, whereas H_0_ finds no such relationship. As an interpretation of the Bayes factors employing Jeffreys’s (1961) classification scheme [28], Bayes factors between 1 and 3 are labelled anecdotal evidence, between 3 and 10 indicate moderate evidence, between 10 and 30 indicate strong evidence, between 30 and 100 indicate very strong evidence, and beyond 100 indicate extreme evidence. The analyses were calculated using JASP [23].

## 3. Results

Half of the participants had their NPE compensation claims approved (50%, *n* = 139). A little over half of the sample were men (57.9%, *n* = 161) and between 30 to 59 years old (52.7%, *n* = 146) (see Table 1). More than half of the participants were inpatients (52.9%, *n* = 152). They were typically diagnosed with mood disorders (56.8%, *n* = 158) or schizophrenia and psychotic disorders (16.2%, *n* = 45).

Concerning the sociodemographic risk factors (age and gender), the frequency distribution of medical errors did not differ as a function of sociodemographic factors (see Table 2).

Concerning clinical risk factors (diagnosis), we found no evidence (BF more than 1—indicative of anecdotal support for H_1_ but the effect was not significant) that the frequency distribution of medical errors differed as a function of clinical factors (see Table 3).

However, the frequency distribution of medical errors differed as a function of institutional factors (type of treatment, patient status, and type of health care). That is, with respect to the type of treatment, apart from the significant *p*-value, the Bayes factor suggested strong evidence for H_1_ compared to H_0_ (see Table 4). As a type of medical error has no inherent order or ranking sequence, post-hoc tests were conducted to explore the relative contribution of the cells by calculating the adjusted residuals of each cell (equivalent to a z-score). Cells that have a standard residual larger than 1.96 were viewed as significant (for *a* = 0.05), but the z-scores were corrected for multiple comparisons (i.e., 12 cells, *p* < 0.004). Post-hoc tests indicated that those who received medication as treatment tended to be proportionally more exposed to an insufficient level of observation (adj. res = 2.91, *p* = 0.004) and those who received medication and psychotherapy as treatment tended to be proportionally more exposed to inadequate treatment, including inadequate medication (adj. res = 3.37, *p* < 0.001). No other cells were significant.

In addition, concerning patient status, apart from the highly significant *p*-value, the Bayes factor suggested extreme evidence for H_1_ compared to H_0_ (see Table 4). Post-hoc tests (z-scores corrected for multiple comparisons, 4 contrast, *p* < 0.0125) indicated that, while medication as treatment was proportionally more prevalent in the inpatient group than among the outpatients (adj. res = 5.54, *p* < 0.0001), inadequate treatment, including inadequate medication was proportionally less prevalent in the inpatient group than the outpatient group (adj. res = 2.84, *p* = 0.004).

Finally, with respect to type of health care, apart from the significant *p*-value, the Bayes factor suggested strong evidence for H_1_ compared to H_0_ (see Table 4). Post-hoc tests (z-scores corrected for multiple comparisons, 4 contrast, *p* < 0.0125) indicated that inadequate treatment, including inadequate medication, was proportionally less prevalent among patients in psychiatric institutions compared to patients at other institutions (adj. res = 3.82, *p* = 0.0001). In addition, inadequate suicide risk assessment was proportionally more prevalent among patients in psychiatric institutions compared to patients at other institutions, but statistical significance did not survive the correction for multiple testing (adj. res = 2.39, *p* = 0.0168).

## 4. Discussion

A main purpose of the current study was to analyse whether the age group, gender, diagnostic category, inpatient/outpatient status, type of institution, and type of treatment received by patients that had died by suicide were associated with different types of medical errors. We found that the patients who had received medication as their main treatment more often were insufficiently observed, whereas patients who had received psychotherapy and medication more often had been provided inadequate treatment, including inadequate medication.

Patients who received medication only as treatment tended to be proportionally more exposed to an insufficient level of observation. This is interesting as systematic observation is particularly important for patients that are undergoing treatment with medication, in order to be able to note any developments in effects and side effects and to observe and address any non-adherence [29,30,31,32]. This is the basis for the recommendation that Norwegian psychiatric units should have clear written observation procedures considering the implementation, adjustment, or cessation of treatment with medications [33]. An insufficient level of observation may be indicative of a poor implementation of the procedures. It is of crucial importance that the units have competent staff (experienced MD/psychiatrist) that have the knowledge needed to prescribe appropriate medical treatment and to evaluate and adjust the treatment according to feedback from the patient and observations by staff. Skilled nursing staff are also needed to uphold good routines in medication dispensing, in communicating with patients, and in making necessary observations [34]. A lack of highly educated and skilled staff is a challenge in some Norwegian institutions, especially outside of the major cities. The results indicate that those who received medication and psychotherapy as treatment tended to be proportionally more exposed to inadequate treatment, including inadequate medication. We lack more detailed information about the medical errors, however, patients that receive psychotherapy are typically treated by clinical psychologists, as this staff group often function as psychotherapists in psychiatric inpatient or outpatient clinics. Importantly, in Norway and in most European countries, clinical psychologists cannot prescribe medications. This implies that patients receiving psychotherapy are prescribed medication by a different staff member (i.e., a medical doctor) than the psychologist who follows the patient most closely. This shared responsibility may open the door for lacking communication and medication errors [35,36]. The medical doctors who share this kind of treatment responsibility may have extensive workloads—which may contribute to the chance of errors occurring. Errors might be prevented by improving routines for interprofessional collaboration in units, by reducing the workload of the medical doctors, thus allowing more time for follow-up, by improving psychopharmacology training of doctors in training, and by increasing prescribing supervision at the unit—for instance, by involving expert pharmacists in the day-to-day treatment of patients [37,38]. Errors relating to the psychotherapeutic treatment itself could be due to factors such as inadequate initial assessment of the patient or inadequate assessment of changes in the patient’s condition, inadequate psychotherapeutic training or lack of supervision of junior staff, the use of psychotherapeutic approaches that do not meet the patient’s needs, lack of time or continuity in treatment, etc. [39,40,41,42]. Many patients have short inpatient stays, which may not allow the time for effective psychotherapeutic treatment [43].

There were no statistically significant findings regarding the patients who received a combination of ECT, medication, and psychotherapy, and errors reported for this group included inadequate medication, treatment, and follow-up, suggesting that patients who receive the most complex treatment may be subject to all types of errors.

Another statistically significant finding was that inadequate treatment, including inadequate medication, was proportionally less prevalent in the inpatient group than in the outpatient group. This may be related to the fact that outpatients are more likely to be treated mainly by other professional groups than medical doctors, including clinical psychologists, psychiatric nurses, and social workers—i.e., professions without specialist knowledge regarding pharmacological treatment.

We also found that inadequate treatment, including inadequate medication, was proportionally less prevalent among patients in psychiatric institutions compared to patients at other institutions. Again, this may be due to the focus of the treatment and the professional group in charge. Medical doctors and psychiatrists with expert psychopharmacological knowledge tend to work in psychiatric institutions, while these specialists are less common in other types of services. Outpatient care may impose additional difficulties in observation and suicide risk assessment because of the therapist’s experience of the lack of control, uncertainty, struggles with ethical issues, poor teamwork, and organisational challenges. Moreover, outpatient care presumes the therapist’s sole responsibility of the patients and assessment of suicide risk assessment and follow-up, which imposes additional challenges in care delivery [44].

The Bayes factors for independence analysis indicated that patients who died by suicide did not tend to differ in terms of sociodemographic and clinical characteristics. Non-significant findings are indicative of either the absence of effect or that the data are simply too insensitive to detect an effect (i.e., small effect size and/or small sample size).

In 2017, the Norwegian Directorate of Health [14] identified some of the weaknesses in the health services that might lead to errors in the assessment and treatment of patients. Areas that were pinpointed were lacking or poor suicide risk assessments, safety measures failures, and the lack of qualified and trained staff. Some specific suggestions made for improvements included improving the quality of suicide assessments and the follow-up of patients at a high risk of suicide and improving individual treatment plans. Furthermore, improving the coordination between treatment levels and units would facilitate the identification of patients with suicidal behaviour and ensure that these patients get access to the health care services they need [14].

Evidence underlying the current recommendations on managing patients with suicidal behaviour at the non-psychiatric institutions is poor, addressing the gap in the clinical practice guidelines [45]. There is a strong need to improve suicide risk assessment at general emergency departments in order to provide adequate treatment or further referral for these patients [45,46]. At the system level, it is necessary to ensure that all cases of suicide are registered, along with the circumstances concerning the suicides. There is a need to systematically analyse the circumstances contributing to treatment failure and to implement improvements in treatment and care that can contribute to reducing the number of individuals who die by suicide during or after the treatment at psychiatric units [14].

One of the strengths of the present study was the inclusion of the Bayes factors for the analyses. Our study can show that a study with high statistical power is indicative of support of the null hypothesis (i.e., an effect too small to matter). Second, according to our best knowledge, this is the first study investigating patients who seek compensation for erroneous treatment that resulted in suicide at health care institutions in Norway. We found no similar studies in the literature, which demonstrates the novelty of the present study. Third, we quantified a documented clinical practice of patient case records and expert assessments into the statistical variables, conveying directly observed challenges that the health care personnel are facing in clinical practice.

The study has several limitations. First, the participants were highly selected, as they were from NPE compensation cases for patients who died by suicide. Patients that had died by suicide and where next-of-kin had not claimed compensation were not included as we lack information about them. Therefore, a possible “selection bias” cannot be excluded. Second, there might be some variables that were relevant that we did not have access to in our data. For instance, we do not have information about the type of psychotherapy and medication the patients received or more precise information about the suicides such as location or other circumstances. Third, while the results suggested that some factors were associated with medical errors, a longitudinal prospective study is needed to exclude alternative explanations for the obtained results. Fourth, our dataset was predefined, which limited our analytic possibilities. It would be recommendable to strategically plan and conduct further data collection on a number of predefined and validated measures.

## 5. Conclusions

The results revealed that patients who received medication as treatment tended to be proportionally more exposed to an insufficient level of observation. Those who received medication and psychotherapy as treatment tended to be proportionally more exposed to inadequate treatment, including inadequate medication. These findings indicate a need to establish observational routines along with enhanced management of medication administration within improved working conditions. Effective communication with these patients is of great importance to improve implementation protocols evaluating the efficacy of the psychotherapeutic treatment. Outpatient units need to establish the teams that will account for challenges during observation and suicide risk assessment and assist the therapists. Units need to account for both optimal training of staff and guidelines for services they provide, while emergency departments require staff with higher competence in suicide risk assessment [47]. At the system level, there is a need to improve the coordination between mental health and other services. Nevertheless, further research is needed to confirm and develop on our findings—especially in the form of longitudinal prospective studies.

## Figures and Tables

**Table 1:** Characteristics of the sample.

Sample Characteristics	Suicide	
	N	%
Age groups		
0–29	90	32.5
30–59	146	52.7
60 and above	41	14.8
Gender		
Male	161	57.9
Female	117	41.2
Diagnosis ICD10		
F20 Schizophrenia and psychotic disorders	45	16.2
F30 Mood (affective) disorders	158	56.8
Other diagnoses	75	27
Patient status		
Inpatient	152	54.7
Outpatient	126	45.3
Type of health care		
Psychiatric institutions	212	76.3
Other institutions	66	23.7
Type of treatment		
Medication only	107	38.5
Medication and psychotherapy	77	27.7
ECT, medication, and psychotherapy	94	33.8
Type of medical error (patient safety incident)		
Insufficient level of observation of symptoms and lack of safety measures	65	23.4
Inadequate suicide risk assessment	88	31.7
Inadequate/delayed diagnostic assessment	82	29.5
Inadequate treatment, including inadequate medication	43	15.5

**Table 2:** Sociodemographic characteristics of patients who had died by suicide.

	Medical Errors (Patient Safety Incidents)				
	ILO (Reference)	ISR	I/DDA	IM/T	
Sample Characteristics	N	N	N	N	Statistics
	%	%	%	%	
Age groups					*χ*^2^(6) = 2.60, *p* = 0.86 BF_10_ = 0.001
0–29	18	28	28	16	
	20	31.1	31.1	17.8	
	Adj.res. = −0.9	Adj. res. = −0.2	Adj. res. = 0.5	Adj. res. = 0.7	
		OR = 1.14	OR = 1.56	OR = 2.44	
		(0.43–3.03)	(0.56–4.33)	(0.65–9.22)	
30–59	36	45	42	23	
	24.7	30.8	28.8	15.8	
	Adj. res. = 0.5	Adj. res. = −0.4	Adj. res. = −0.2	Adj. res. = 0.1	
		OR = 0.92	OR = 1.17	OR = 1.76	
		(0.38–2.24)	(0.45–3)	(0.50–6.18)	
60 and above	11	15	11	4	
	26.8	36.6	26.8	9.8	
(Reference)	Adj. res. = 0.6	Adj. res. = 0.7	Adj. res. = −0.4	Adj. res. = −1.1	
Gender					*χ*^2^(3) = 1.78 *p* = 0.062 BF_10_ = 0.046
Male	38	48	52	23	
	23.6	29.8	32.3	14.3	
	Adj. res. = 0.1	Adj. res. = −0.8	Adj. res. = 1.2	Adj. res. = −0.6	
Female	27	40	30	20	
	23.1	34.2	25.6	17.1	
	Adj. res. = −0.1	Adj. res. = 0.8	Adj. res. = −1.2	Adj. res. = 0.6	
(Reference)		OR = 0.85	OR = 1.23	OR = 0.82	
		(0.45–1.63)	(0.63–2.40)	(0.38–1.78)	

ILO—Insufficient level of observation of symptoms and lack of safety measures. ISR—Inadequate suicide risk assessment. I/DDA—Insufficient/delayed diagnostic assessment. IM/T—Inadequate treatment, including inadequate medication.

**Table 3:** Clinical characteristics of patients who died by suicide.

	Medical Errors (Patient Safety Incidents)				
	ILO (Reference)	ISR	I/DDA	IM/T	
	N	N	N	N	Statistics
	%	%	%	%	
Diagnosis					*χ*^2^(6) = 6.08 *p* = 0.41 BF_10_ = 0.008
F20 Schizophrenia and psychotic dis.	11	10	19	5	
	24.4	22.2	42.2	11.1	
	Adj. res. = 0.2	Adj. res. = −1.5	Adj. res. = 2	Adj. res. = −0.9	
		OR = 0.744	OR = 1.48	OR = 0.58	
		(0.26–2.14)	(0.56–3.92)	(0.16–2.08)	
F30 Mood (affective) dis.	36	56	42	24	
	22.8	35.4	26.6	15.2	
	Adj. res. = −0.3	Adj. res. = 1.6	Adj. res. = −1.2	Adj. res. = −0.1	
		OR = 1.27	OR = 1.00	OR = 0.86	
		(0.60–2.70)	(0.46–2.16)	(0.36–2.04)	
Other diagnoses (Reference)	18	22	21	14	
	24.0	29.3	28.0	18.7	
	Adj. res. = 0.1	Adj. res. = −0.5	Adj. res. = −0.3	Adj. res. = 0.9	

**Table 4:** Institutional characteristics of patients who died by suicide.

	Medical Errors (Patient Safety Incidents)				
	ILO (Reference)	ISR	I/DDA	IM/T	
	N	N	N	N	Statistics
	%	%	%	%	
Type of treatment					*χ*^2^(6) = 21.80 *p* ˂ 0.001 BF_10_ = 30.74
Medication	35	29	28	15	
	32.7	27.1	26.2	14.0	
	Adj. res. = 2.9	Adj. res. = −1.3	Adj. res. = −1	Adj. res. = −0.5	
		OR = 0.37	OR = 0.37	OR = 0.98	
		(0.17–0.79)	(0.17–0.79)	(0.33–2.87)	
Med + psychoth	14	23	19	21	
	18.2	29.9	24.7	27.3	
	Adj. res. = −1.3	Adj. res. = −0.4	Adj. res. = −1.1	Adj. res. = 3.4	
		OR = 0.73	OR = 0.62	OR = 3.43	
		(0.30–1.77)	(0.25–1.54)	(1.12–10.47)	
ECT + med + psychoth (Reference)	16	36	35	7	
	17.0	38.3	37.2	7.4	
	Adj. res. = −1.8	Adj. res. = 1.7	Adj. res. = 2	Adj. res. = −2.9	
Patient status					*χ*^2^(3) = 36.68 *p* ˂ 0.001 BF_10_ = 1.246 × 10^6^
Inpatient	55	45	37	15	
	36.2	29.6	24.3	9.9	
	Adj. res. = 5.5	Adj. res. = −0.8	Adj. res. = −2.1	Adj. res. = −2.8	
Outpatient	10	43	45	28	
	7.9	34.1	35.7	22.2	
	Adj. res. = −5.5	Adj. res. = 0.8	Adj. res. = 2.1	Adj. res. = 2.8	
(Reference)		OR = 1.90	OR = 0.15	OR = 0.10	
		(0.09–0.42)	(0.07–0.33)	(0.04–0.24)	
Type of health care					*χ*^2^(3) = 15.12 *p* ˂ 0.001 BF_10_ = 21.80
Psychiatric institution	51	75	63	23	
	24.1	35.4	29.7	10.8	
	Adj. res. = 0.5	Adj. res. = 2.4	Adj. res. = 0.1	Adj. res. = −3.8	
Other institution	14	13	19	20	
	21.2	19.7	28.8	30.3	
	Adj. res. = −0.5	Adj. res. = −2.4	Adj. res. = −0.1	Adj. res. = 3.8	
(Reference)		OR = 1.58	OR = 0.91	OR = 0.32	
		(0.69–3.65)	(0.42–1.99)	(0.14–0.73)	

## Data Availability

The data are not publicly available due to privacy restrictions, but may be obtained from the authors on reasonable request.
